# Down for the count: *Cryptosporidium* infection depletes the gut microbiome in Coquerel’s sifakas

**DOI:** 10.1080/16512235.2017.1335165

**Published:** 2017-06-15

**Authors:** Erin A. McKenney, Lydia K. Greene, Christine M. Drea, Anne D. Yoder

**Affiliations:** ^a^ Department of Biology, Duke University, Durham, NC, USA; ^b^ University Program in Ecology, Duke University, Durham, NC, USA; ^c^ Department of Evolutionary Anthropology, Duke University, Durham, NC; ^d^ Duke Lemur Center, Durham, NC, USA

**Keywords:** Enteric infection, gut microbiota, lemur, protozoan pathogen, strepsirrhine primate

## Abstract

**Background**: The gut microbiome (GMB) is the first line of defense against enteric pathogens, which are a leading cause of disease and mortality worldwide. One such pathogen, the protozoan *Cryptosporidium*, causes a variety of digestive disorders that can be devastating and even lethal. The Coquerel’s sifaka (*Propithecus coquereli*) – an endangered, folivorous primate endemic to Madagascar – is precariously susceptible to cryptosporidiosis under captive conditions. If left untreated, infection can rapidly advance to morbidity and death.

**Objective**: To gain a richer understanding of the pathophysiology of this pathogen while also improving captive management of endangered species, we examine the impact of cryptosporidiosis on the GMB of a flagship species known to experience a debilitating disease state upon infection.

**Design**: Using 16S sequencing of DNA extracted from sifaka fecal samples, we compared the microbial communities of healthy sifakas to those of infected individuals, across infection and recovery periods.

**Results**: Over the course of infection, we found that the sifaka GMB responds with decreased microbial diversity and increased community dissimilarity. Compared to the GMB of unaffected individuals, as well as during pre-infection and recovery periods, the GMB during active infection was enriched for microbial taxa associated with dysbiosis and rapid transit time. Time to recovery was inversely related to age, with young animals being slowest to recover GMB diversity and full community membership. Antimicrobial treatment during infection caused a significant depletion in GMB diversity.

**Conclusions**: Although individual sifakas show unique trajectories of microbial loss and recolonization in response to infection, recovering sifakas exhibit remarkably consistent patterns, similar to initial community assembly of the GMB in infants. This observation, in particular, provides biological insight into the rules by which the GMB recovers from the disease state. Fecal transfaunation may prove effective in restoring a healthy GMB in animals with specialized diets.

## Introduction

The gut microbiome (GMB) is the complex community of bacteria, archaea, eukaryotes, and their respective genomes, that inhabit animal gastrointestinal tracts. A healthy or symbiotic GMB aids in digestion [1,2], produces critical nutrients [3,4], and interacts dynamically with the immune system [5]. Healthy GMBs also resist pathogens by producing bacteriocins, sequestering niche space and resources, and/or activating host immune defenses [6]. GMBs also have been implicated in diarrheal infections caused by bacteria (*Vibrio cholera* [7,8], *Escherichia coli* [9], *Salmonella* [10]) and protozoans (*Giardia* [11], *Cryptosporidium* [12,13]). For example, compared to conventional controls, mice with a normal immune system, but low GMB complexity, were unable to clear *Salmonella* pathogens from the lumen [14]. In some cases, the GMB recovers from enteric infection to a new stable state [15]. This shift possibly reflects stochasticity, but also could result from hosts selecting for particular taxa or functions that confer resistance. Unfortunately, in most studies of the GMB and enteric infection, samples are primarily collected after infections occur. Thus, examination of the pre-infected GMB is limited (although see David et al. [16]). Thus, we lack specific understanding of what constitutes a sufficiently healthy GMB to confer resistance, facilitate clearance, and maintain gut homeostasis.

*Cryptosporidium* is a particularly problematic gastrointestinal parasite. Present worldwide, this pathogenic protozoan infects a diverse range of vertebrates, including humans [17]. Oocysts can be transmitted via the fecal–oral route, are often present in groundwater, and can survive for long periods under myriad environmental conditions [18]. Nevertheless, cryptosporidiosis is generally most common in warm and moist environments or climates. The pathogen’s life cycle is completed within a single host; replication cannot occur outside hosts [17]. After ingestion, each oocyst that reaches the stomach and small intestine bursts to release four motile sporozoites. Sporozoites invade host gastrointestinal epithelial cells, where they reproduce to form new oocysts. Infected individuals typically present with symptoms of watery diarrhea owing to increased intestinal permeability, chloride secretion, and malabsorption, all of which are related to the host’s immune response to infection [19,20]. In humans, children under 5 years old are most vulnerable to infection, owing to their lack of protective immunity and increased fecal–oral transmission [21]. For immunosuppressed patients, such as those with human immunodeficiency virus/acquired immune deficiency syndrome (HIV/AIDS), *Cryptosporidium* infection can be fatal [22].

The relationship between cryptosporidiosis and the GMB is garnering increased research attention, with laboratory rodents often serving as model organisms. Rodents provide a tractable system for testing specific pathogenic mechanisms and interactions with the GMB. For instance, immunosuppressed mice that are germ free are significantly less resistant to *Cryptosporidium* oocysts than are controls [12]. Moreover, probiotic *Lactobacillus* and *Bifidobacterium* species may help immunosuppressed mice to resist cryptosporidiosis [23,24]. Although rodent models offer advantages in terms of large sample sizes, experimental control, and reproducibility, their phylogenetic distance potentially reduces their biological relevance as models for understanding human disease. Thus, we stand to gain a better understanding of the resistant GMB and the means for combating destructive pathogens by studying the links between cryptosporidiosis and the structure of the GMB, across the course of infection, in a variety of host taxa that include primate models.

**Figure 1.**
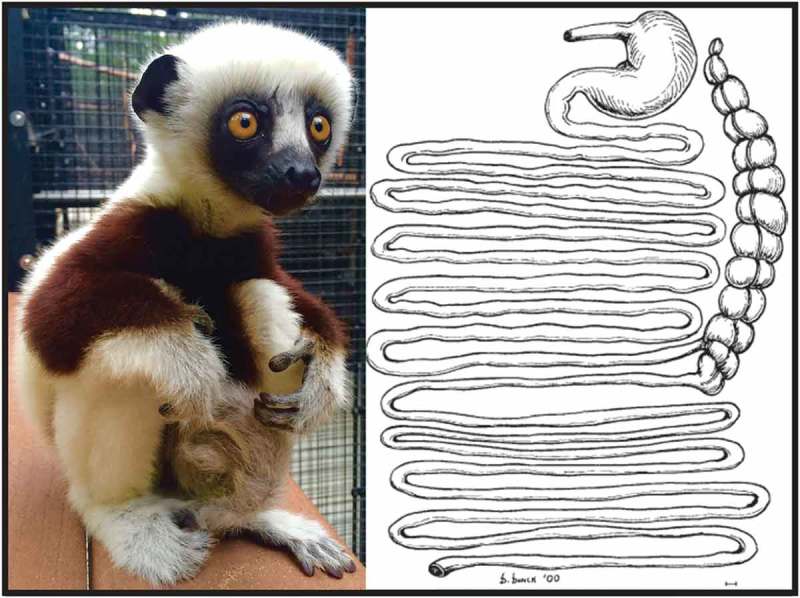
(a) Photograph of a young Coquerel’s sifaka at the Duke Lemur Center; (b) drawing of the gastrointestinal tract of a Coquerel’s sifaka, reproduced with permission from John Wiley and Sons from [27].

As a consumer of a diverse folivorous diet [25] that requires specialized gastrointestinal morphology, the sifaka is a novel primate model in which to probe the links between GMB structure and enteric infection. Compared to other lemur species, the sifaka’s gastrointestinal morphology includes significantly elongated intestines, an enlarged cecum, and longer gut transit time [26,27] (Figure 1), to allow adequate microbial fermentation of dietary fiber. The sifaka GMB is likewise specialized compared to that of other lemurs: sifaka GMBs are enriched for plant-degrading bacteria (e.g. *Ruminococcus*sp.) and genes (e.g. tanases) and, although tightly conserved across individuals, are more diverse than those of other lemurs [28] (McKenney et al. in prep.). In the wild, the structure of sifaka GMBs varies seasonally and corresponds to shifts in nutrient availability [29].

Of the nine critically endangered sifaka species endemic to Madagascar, the Coquerel’s sifaka (*Propithecus coquereli*) is the only one that is successfully kept in captivity. Nevertheless, it remains disposed towards fragile gut health and is uniquely susceptible to infection with protozoan pathogens, including *Cryptosporidium parvum* [19]. Captive sifakas living in North America manifest cryptosporidiosis similarly to humans, with symptoms including lethargy, anorexia, and fulminating diarrhea, which can be prolonged (> 1 week) or persistent (> 2 weeks), chronic, severe, and even fatal. Infant and subadult sifakas are most susceptible, with adults having infrequent and less severe infections [19]. Chronic cryptosporidiosis is complicated by malabsorption and malnutrition, which are of particular concern for folivores that depend on their gut microbes to digest high-fiber, leaf-based diets. Thus, in addition to revealing comparative insights into primate gastrointestinal disease states, an understanding of how the sifaka GMB reacts to *Cryptosporidium* infection can offer insight into preventive healthcare measures and treatment options for this endangered primate. Moreover, lemurs share a close evolutionary history with humans and are already proving to be useful models in studies of human disease [30,31]. In humans, diverse diet, lifestyle, and medical treatment choices introduce variation to the microbiome and can thus complicate rigorous and controlled studies. In turn, this makes it difficult to differentiate between changes that result specifically from dysbiosis (i.e. symptom) and those that result from disease (i.e. cause). By contrast, sifakas’ consistent high-fiber diet and lifestyle in captivity, as well as their elongated gut, all select for complex microbial membership with minimal variation either within or between healthy individuals [28]. As a biological model, the sifaka is thus useful for assessing microbial dynamics and host regulation during various perturbations and recovery.

Here, we examine the GMB of captive Coquerel’s sifakas across seasonal bouts of cryptosporidiosis, analyzed as three diagnosable states: pre-infection, infection, and recovery periods. We use statistical tests to detect the community shifts associated with infectious perturbation and determine which host metadata and microbial features might contribute to pathogen resistance and recovery from infection. Ultimately, these data can be used to contribute to the design of prebiotic or probiotic therapies for captive lemurs, as well as inform treatment options for humans with enteric disease.

### State 1: pre-infection

If a stable, symbiotic microbiome underlies resistance to pathogen colonization, we would expect that the GMB of ‘unaffected’ individuals (i.e. those that did not contract an infection) would differ in diversity or taxonomic structure from that of ‘pre-infection’ individuals (i.e. those that eventually contracted an infection). If, however, a microbial deficit does not make some individuals more susceptible to infection than others, we would expect the GMB of unaffected and pre-infection individuals to be indistinguishable.

### State 2: infection

During active infection and diarrheal shedding, we expect GMB diversity to decline and the taxa present to shift towards microbes tolerant of oxygen and/or those typically associated with dysbiosis [32]. Community alterations may include increased proportions of pre-existing taxa or introduction of previously absent taxa, which are opportunists adapted to features (e.g. more rapid transit times) associated with a disrupted environment.

### State 3: recovery

We predict that recovery of the GMB through secondary colonization will mimic that of initial colonization [28]: microbial diversity should increase across recovery, and the taxonomic structure of the GMB should return to a stable community similar to that of either pre-infection or unaffected individuals [33]. These patterns are likely to be most evident in younger animals, because infant sifakas are known to have a less robust GMB than adults [28].

## Materials and methods

### Subjects and housing

The subjects were 35 captive Coquerel’s sifakas (19 female, 16 male) that ranged in age from 0.5 to 25 years. They were housed at the Duke Lemur Center (DLC) in Durham, NC, USA, in 10 mixed-sex social groups, each of which comprised a dominant breeding pair and associated offspring or relatives. Habitually, each social group occupies its own large (146 m^2^/animal) indoor/outdoor pen year round. Six of the groups gain additional access to large, forested enclosures (0.6–5.8 ha), in which they semi-free range when ambient temperatures remain above 5 ºC (41 ºF). Throughout the year, the subjects are fed a once-daily diet of Leaf-Eater Primate Diet Mini-Biscuit (No. 5672, Mazuri, Brentwood, MO) accompanied by fresh vegetables, greens, beans, nuts, and freshly cut leaves from local flora. When semi-free ranging, the subjects are able to forage on additional local vegetation. Water is always freely available. All of the subjects are individually identifiable via colored collars, tail shaves, and distinguishing markings.

The DLC animals are maintained in accordance with the US Department of Agriculture regulations and the National Institutes of Health Guide for the Care and Use of Laboratory Animals. The research protocols for this study were approved by the Institutional Animal Care and Use Committee (IACUC) of Duke University (protocol number A171-09-06).

### Study period and *Cryptosporidium* detection

We conducted the study across a 3 year period from 2013 to 2016. During this time, nine sifakas contracted *Cryptosporidium* infections during late spring or early summer and only one individual became infected in the winter (Table 1); there were no *Cryptosporidium*-related mortalities during the 3 year study period. The majority of infected individuals were either infants (*n* = 2) or subadults (*n* = 5); only three were aged 5 years or older. As far as possible, the husbandry practices implemented at the DLC are specifically designed to prevent infection with *Cryptosporidium*, thus limiting the available sample size of infected individuals. In addition, antimicrobial treatments and subsequent fecal microbiome transplants may be administered in cases with a severe prognosis (Table 1). These husbandry practices also guarantee that animals are, otherwise, in optimal health, allowing us to clearly delineate the impacts of infection versus other possible causes of morbidity.

**Table 1.** Metadata per affected sifaka, presented in order of ascending age.

Subject	Age (years)	Sex	Social group	Study year	Illness duration (days)^a^	Antimicrobials (treatment duration, days)	Fecal transplant
Beatrice^b^	1	F	M	2013	11	Azithromycin (1), nitazoxanide (3)	No
Valeria	1	F	A	2015	22	Ampicillin (3), ceftazidime (3), nitazoxanide (10)	Yes
Gisela	2	F	G	2013	15	Ampicillin (5), ceftazidime (5), ceftiofur (5), metronidazole (5), nitazoxanide (9)	Yes
Remus	2	M	R	2013	20	Ampicillin (10), ceftazidime (10), metronidazole (6), nitazoxanide (19)	Yes
Aemilia	2	F	D	2015	9	No	No
Gertrude^c^	3	F	P	2016	> 6	No	No
Pontius	3	M	D	2015	8	Metronidazole (5)	No
Arcadia	5	F	D	2015	13	No	No
Rupilia	14	F	R	2013	> 8	No	No
Antonia^d^	18	F	A	2015	> 3	Amoxicillin (5), ampicillin (3)	No

^a^ Values preceded by a greater than symbol (>) are estimates; first day of infection is unknown for these subjects (Gertrude, Rupilia, and Antonia).

^b^ Only pre-infection and recovery samples were available.

^c^ Infection manifested in winter 2016.

^d^ On antimicrobials only during recovery, because they were administered to treat a separate injury, not the infection.

*Cryptosporidium* infections are detected by DLC veterinarians using acid-fast staining, as described previously [19,34]. In brief, fecal samples are collected from those sifakas showing symptoms of infection, including lethargy, anorexia, or diarrhea. The samples are prepared for microscopic analysis using a concentrated formalin–ethyl acetate method, and smears are prepared and stained using a modified acid-fast technique. A positive test confirms an active infection with *Cryptosporidium*, including the presence of oocysts. The infected sifakas are continuously monitored for oocysts across the period of infection and are deemed recovered (free of infection) after three consecutive negative smears.

### Fecal sampling

Although fecal bacteria may not accurately represent mucosal communities [35], fecal collection is appreciably non-invasive compared to biopsy sampling and is, thus, both more feasible in studies of endangered animals and most comparable to the majority of human studies. Sterile techniques were used in fecal sampling. We collected only fresh fecal samples, post-voiding, placing the sample directly into sterile tubes using sterile wooden spatulas. We immediately placed the tubes on ice and stored them at −80 °C within 1 h of collection. Because *Cryptosporidium* infection at the DLC primarily occurs in late spring [19], we began collecting fecal samples from all of the sifakas between late winter (February) and early spring (May). Because cryptosporidiosis, although more prevalent in younger animals, can affect sifakas at any age, we wanted to establish a ‘healthy’ profile for individuals of all ages. For individuals that eventually became infected, these samples constituted pre-infection samples from which we could both gauge their diagnostic potential and assess the impact of intestinal disease on the GMB. As no individuals became infected in year 2, we only included samples from unaffected adults in years 1 and 3. Across these two study years, we ultimately collected 50 samples from the 35 unaffected individuals that did not become infected during our study period. Collecting duplicate samples from unaffected individuals across both study years also allowed us to better control for any biases in the data due to study year or extraction kit. For sifakas that contracted cryptosporidiosis, we endeavored to sample them all before infection, at least weekly across the period of infection, and weekly for 2 months after infections had cleared. When possible, we collected samples more frequently (e.g. daily) across the period of infection.

### DNA extraction and genetic analyses

We extracted genomic DNA (gDNA) from fecal samples for downstream genetic analyses using commercially available extraction kits. The first batch of samples, including those from 20 unaffected individuals in year 1 and from six affected individuals in both years, were extracted using the QIAamp® DNA Stool Mini Kit (QIAGEN, Hilden, Germany), whereas the second batch, including samples from 30 unaffected and six affected individuals in year 3, were extracted using the PowerSoil® DNA Isolation Kit (Mo Bio, Carlsbad, CA, USA). Although different extraction protocols can ultimately influence the sequence data generated [36], these two extraction kits have been shown to produce similar results on microbial community composition [37]. Both extraction protocols use a similar procedure involving cell lysis, gDNA purification, and the retention of gDNA on silica membranes. Whereas the PowerSoil kit uses a combination of chemical and mechanical lysis, the QIAamp kit relies on only chemical lysis; we therefore added a mechanical lysis step (vortex with silica beads). For both extraction protocols, we used 0.1–0.2 g of frozen feces and followed the manufacturers’ specifications, adding a heat-blocking step before bead-beating. For the QIAamp kit, the samples were heated to 95 °C for 5 min; for the PowerSoil kit, the samples were heated to 65 °C for 10 min. The concentration of extracted gDNA was determined using a Qubit™ 3.0 fluorometer (Thermo Fisher Scientific, Waltham, MA, USA).

We shipped aliquots of gDNA, overnight on dry ice, in two batches (year 1 and year 3) to Argonne National Laboratories (Lemont, IL, USA) for sequencing of the 16S ribosomal RNA gene. We used the 515F (GTG-CCA-GCM-GCC-GCG-GTA-A) and 806R (GGA-CTA-CHV-GGG-TWT-CTA-AT) primers to sequence the v4 variable region. All sequence data are available in the NCBI Sequence Read Archive under accession numbers SAMN06349027–SAMN06349170.

### Bioinformatic and statistical analyses

We processed the sequence data using Quantitative Insights into Microbial Ecology (QIIME version 1.9.1) [38]. All scripts necessary to reproduce the analytical workflow are available in **Supplemental File 1**. Specifically, forward and reverse reads were joined together using ea-utils [39], quality filtered, and demultiplexed per individual using default parameters, as described previously [28]. We processed raw reads from the two sequencing runs separately, then concatenated the resulting FASTA files and picked operational taxonomic units (OTUs) from the complete data set based on 97% sequence identity, using the *de novo* UClust method [40]. Taxonomy of OTUs was assigned using the summarize.taxa.py function in QIIME (**see Supplemental File 1**), which blasts sequences against the Greengenes database (version 13_8).

We calculated the unique fraction or UniFrac distance metric [41] to measure the phylogenetic distance between samples, weighted by the relative abundance of each bacterial lineage detected per community. We used a distance matrix to calculate principal coordinates analyses (PCoA) and pairwise comparisons between health-status groups. Whereas PCoA plots detect relationships between community composition, individual, and health status, we assessed pairwise GMB distance with Student’s *t* tests and Bonferroni corrections to quantify community disruption relative to health status. We also calculated linear discriminant analysis effect size [42] to determine which significantly enriched OTUs drove the observed alpha and beta diversity dynamics across individuals during health, infection, and recovery. Lastly, we calculated four metrics of alpha diversity (i.e. within-sample diversity) on all samples using the QIIME software: Good’s coverage, the Shannon and Simpson indices, and Faith’s phylogenetic diversity (PD) [43]. We retained all of the samples, as Good’s coverage was 95% or greater for every sample. We report Good’s coverage as a quality measure, but we completed statistical analyses for the remaining three metrics.

To assess the influence of *Cryptosporidium* infection on alpha diversity, we ran three suites of linear mixed models using the glmmADMB package (version 0.8.3.3 [44]) in Rstudio (version 0.99.902) [45]. In all models, we used as the response variable the Shannon index, Simpson index, or the log(PD) (to better normalize the data). We always included the individual sifaka and study year (two classes: one and three) as random variables. The data for Shannon, Simpson, and log(PD) most closely resembled the normal distribution: We therefore used the Gaussian family for all models. For each model included herein, we determined the best fit via stepwise deletion, removing the variable with the highest *p* value, and refitting the model until only significant explanatory variables (*p* < 0.05) remained. We added each non-significant variable back into the model one by one to ensure that we did not overlook any significant effects. We present each full model below.

**Model 1**

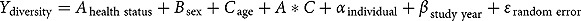

We first determined whether the alpha diversity of samples from pre-infection individuals was equal to that of samples from unaffected individuals. In these models, we included health status (two classes: pre-infection and unaffected), host sex (two classes: female and male), host age (continuous variable, in years), and the interaction between health status and age, as explanatory variables.

**Model 2**

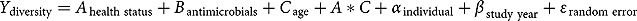

We next asked whether alpha diversity varied with health status across only the 10 sifakas that contracted *Cryptosporidium* infection. Because only two males of similar age became infected, we could not reliably test for sex differences among infected individuals. We therefore did not include host sex in these analyses. In this second set of models, we included as explanatory variables health status (three classes: pre-infection, infection, and recovery), host age (continuous variable, in years), concurrent antimicrobial use (two classes: yes and no), and the interaction between health status and age.

**Model 3**

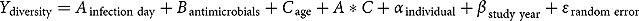

**Figure 2.**
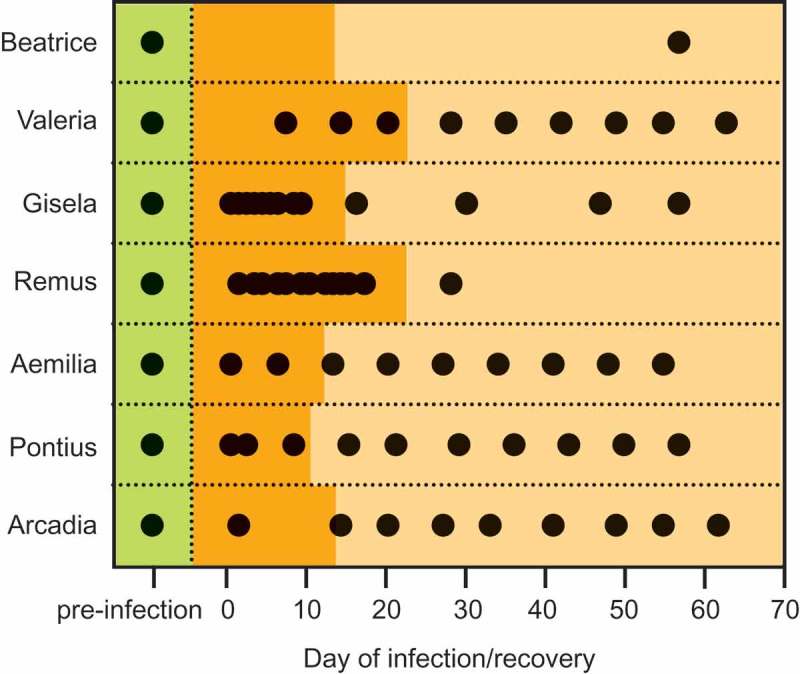
Schematic illustrating the timing of fecal sample collection relative to infection day across the study for all individuals whose initial day of infection was known, including during pre-infection (green), active infection (dark orange), and recovery (light orange) .

Lastly, we examined diversity within infected individuals to determine how alpha diversity changed across the period of infection. In these models, we excluded three infected individuals (two adults, one subadult) for which we could not conclusively determine the initial day of infection. For the remaining seven individuals (Figure 2), our models included as explanatory variables age class (continuous variable, in years), infection day (continuous variable, in days), concurrent antibiotic use (two classes: yes and no), and the interaction between infection day and age.

## Results

### Model 1: the gut microbiome before infection

**Figure 3.**
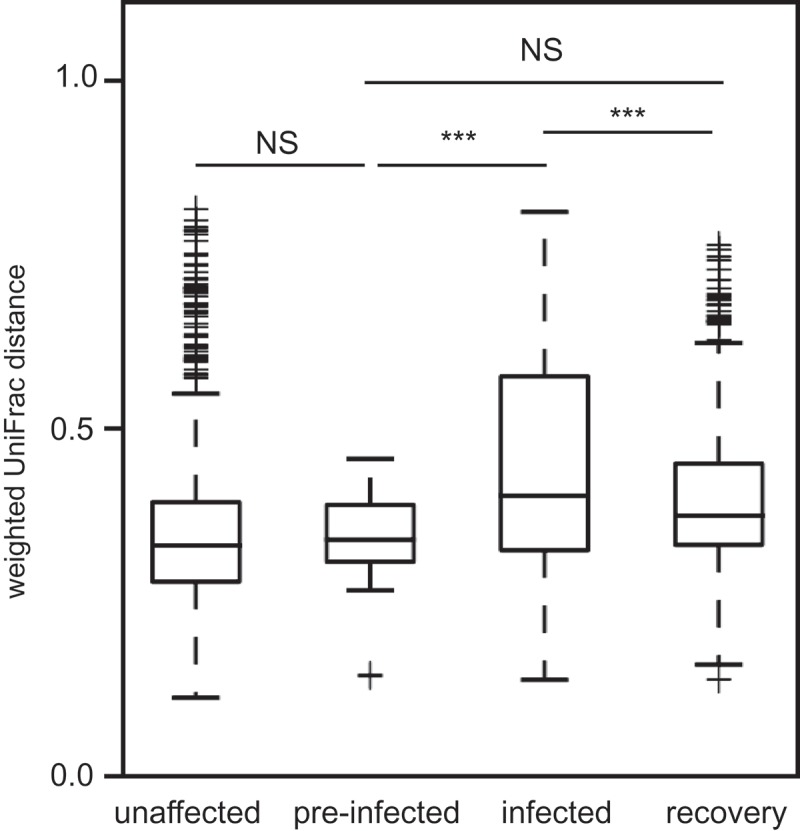
Boxplots of mean ± quartiles weighted UniFrac distance for all pairwise comparisons within health status, including unaffected, pre-infection, infection, and recovery pairs. NS=non-significant result; ***p < 0.001.

Before infection, the sifakas that eventually contracted *Cryptosporidium* hosted GMBs that were similar to those of unaffected adults. Alpha diversity measures did not vary between unaffected and pre-infected individuals, as captured by the Shannon, Simpson, or PD indices (Table 2), and weighted UniFrac distances were similar between all unaffected and pre-infected sample pairs (*t* = 0.80, df = 50, *p* = 1.0) (Figure 3).

**Table 2.** Alpha diversity in fecal samples from unaffected versus pre-infection subjects.

		Shannon		Simpson		PD	
Explanatory variable	Trend	*z*	*p*	*z*	*p*	*z*	*p*
Health status	Unaffected = pre-infection	−0.98	0.32	−0.83	0.41	−0.69	0.49
Age	No trend	−0.67	0.50	−0.93	0.35	−0.38	0.71
Sex	Female = male	−0.85	0.40	0.81	0.42	−0.94	0.35
Health status * Age	No trend	0.73	0.47	0.56	0.58	1.30	0.19

PD = phylogenetic diversity.

### Model 2: the gut microbiome across health statuses

Host age and health status, as well as their interaction, were significantly associated with GMB alpha diversity (Table 3) when comparing only the samples obtained from those sifakas that contracted infection. These subjects tended to host greater microbial diversity before than during infection, as captured by the Simpson index. Overall, age was not associated with GMB diversity; however, the interaction between age and health status indicates that younger animals, compared to their older counterparts, had significantly reduced microbial diversity during recovery. Not surprisingly, concurrent antimicrobial use (Tables 1 and 3) was associated with a significant and dramatic decrease in GMB diversity. Moreover, recovery was associated with a significantly more diverse GMB than during infection, as captured by both the Shannon and Simpson indices. Recovery was especially pronounced for the subjects treated with antimicrobials, each of which subsequently received a fecal microbiome transplant.

**Table 3.** Alpha diversity across health statuses for all infected sifakas.

		Shannon		Simpson		PD	
Explanatory variable	Trend	*z*	*p*	*z*	*p*	*z*	*p*
Health status	Pre-infection (>) infection	0.47	0.64	1.70	0.09	−0.35	0.73
Health status	Infection < recovery	**2.35**	**0.019**	**2.78**	**0.0054**	1.21	0.23
Age	No trend	1.42	0.157	1.31	0.1890	0.49	0.63
Antimicrobials	No > yes	**−6.87**	**< 0.001**	**−5.86**	**< 0.001**	**−7.57**	**< 0.001**
Health status * Age	Younger sifakas < older sifakas in recovery	**−1.96**	**0.05**	**−3.09**	**0.002**	−0.84	0.40

PD = phylogenetic diversity.

Significant results are shown in bold.

**Figure 4.**
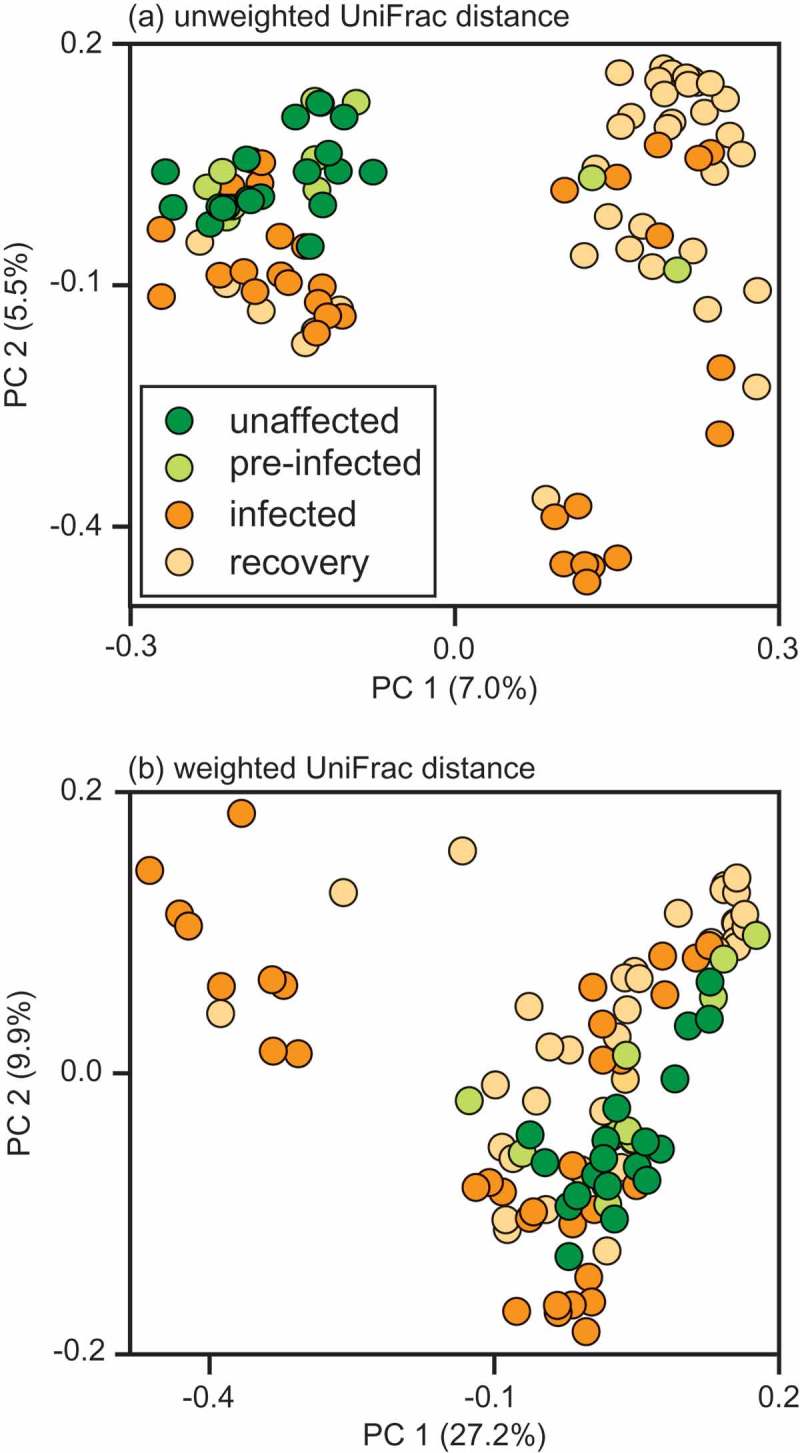
Principal coordinates (PC) analysis plots of (a) unweighted and (b) weighted UniFrac distances for all samples, including those collected from unaffected sifakas, as well as those from sifakas during pre-infection, active infection, and recovery periods.

Weighted and unweighted UniFrac distances further revealed infection to be associated with community disruption, as shown by box and PCoA plots (Figures 3 and 4). Whereas variation between sample pairs in pre-infection and recovery periods was similar (*t* = −3.10, df = 47, *p* < 0.14), samples collected during infection exhibited significantly greater variation than did samples from pre-infection (*t* = 3.63, df = 46, *p *< 0.001) or recovery (*t* = 3.48, df = 72, *p* < 0.001) periods, probably reflecting microbial community disturbance during infection. We used linear discriminant analysis to identify the taxa that were significantly enriched at each health status (Table 4). Healthy (unaffected and pre-infection) samples were distinguished from infection and recovery by 10 biomarkers, infection was distinguished by six biomarkers, and recovery by just four biomarkers.

**Table 4.** Bacterial taxa that were significantly enriched during the different health statuses of sifakas, as revealed by linear discriminant analysis effect size (LEfSe) analysis [42].

Health status	Phylum	Order	Family	Genus	Log(LDA)
Healthy (unaffected and pre-infection)	Actinobacteria	Bifidobacteriales	Bifidobacteriaceae	*Bifidobacterium*	3.74
	Bacteroidetes	Bacteroidales	Rikenellaceae	Unknown	4.45
				Other	2.40
	Firmicutes	Clostridiales	unknown	Unknown	4.49
			Mogibacteriaceae	*Anaerovorax*	3.22
			Gracilibacteraceae	Unknown	3.68
			Lachnospiraceae	*Butyrivibrio*	2.97
				Other	3.66
	Proteobacteria	Aeromondales	Succinivibrionaceae	*Succinivibrio*	2.79
	Verrucomicrobia	Verrucomicrobiales	Verrucomicrobiaceae	*Akkermansia*	3.40
Infection	Firmicutes	Lactobacillales	Enterococcaceae	*Enterococcus*	3.06
		Turicibacterales	Turicibacteraceae	*Turibacter*	2.47
		Clostridiales	Peptococcaceae	Unknown	2.39
			Other	Other	3.14
	Proteobacteria	Desulfovibrionales	Desulfovibrionaceae	*Desulfovibrio*	3.65
		Enterbacteriales	Enterbacteriaceae	Unknown	3.83
Recovery	Bacteroidetes	Bacteroidales	Unknown	Unknown	4.35
	Firmicutes	Clostridiales	Clostridiaceae	Unknown	3.46
			Lachnospiraceae	Unknown	4.28
		Erysipelotrichales	Erysipelotrichaceae	*Coprobacillus*	3.06

Significance values are *p* < 0.01 for all taxa.

**Figure 5.**
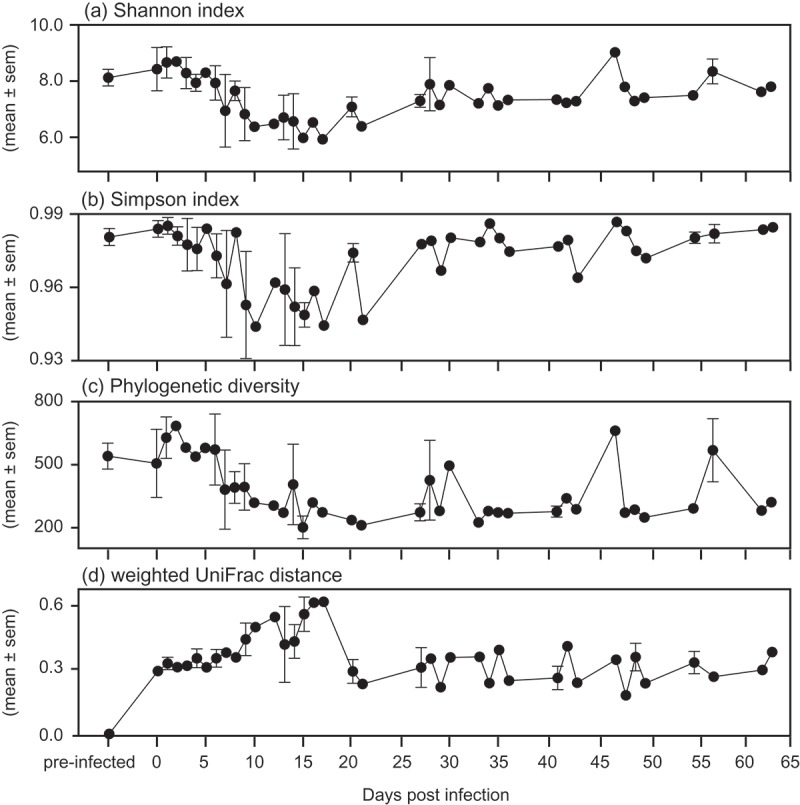
Mean ± standard error (sem) alpha and beta diversity metrics relative to infection day across the study for all of the sifakas whose initial day of infection was known. (a) Shannon index, (b) Simpson index, (c) phylogenetic diversity, and (d) weighted UniFrac distance from pre-infection (i.e. baseline).

**Figure 6.**
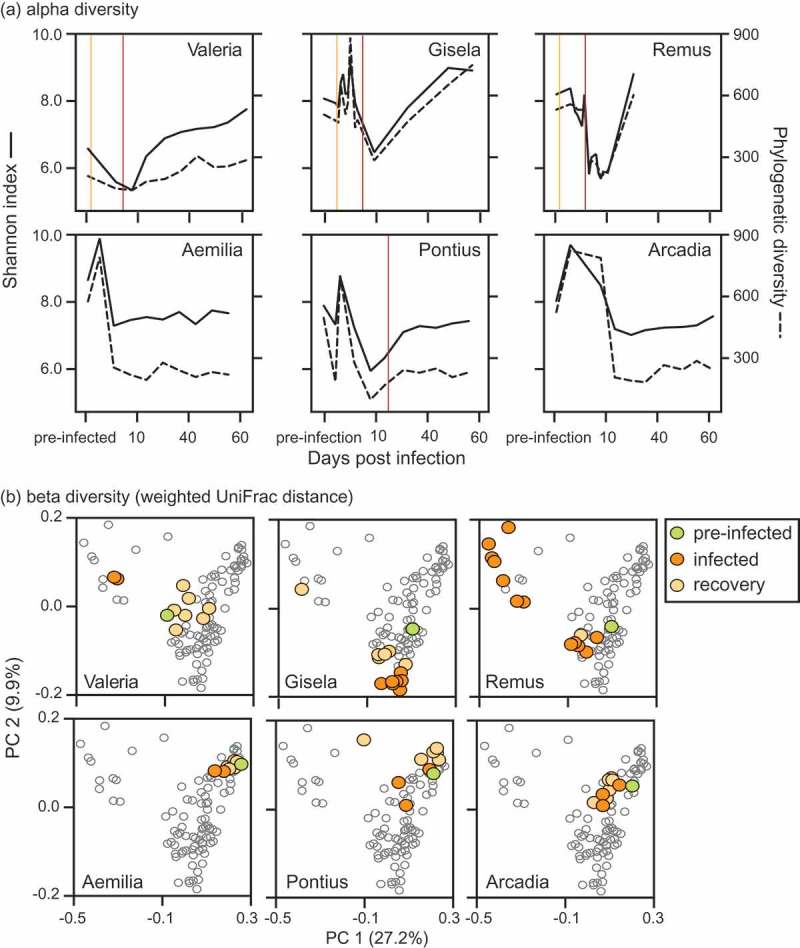
Trajectories for individual sifakas during infection and recovery relative to measures of gut microbial (a) alpha and (b) beta diversity. (a) Shannon index (solid lines) incorporates species richness and evenness, whereas phylogenetic diversity (dashed lines) quantifies taxonomic relatedness across communities. Vertical lines mark the onset of antimicrobial (orange) and antibiotic (red) treatments. (b) Principal coordinates analysis plots of UniFrac distance, weighted by relative abundance.

When considering only the subjects for which we could identify the initial day of infection (i.e. day 0), the number of days post-infection was, or tended to be, significantly and positively associated with GMB alpha diversity (Table 5). Specifically, after an initial, relatively steep decrease in diversity at the onset of infection, the Shannon, Simpson, and PD indices increased gradually across recovery (Figure 5(a)–(c)). We next plotted the UniFrac distance of infection and recovery samples from the pre-infection baseline for each individual, to measure the community disruption across the number of days post-infection (Figure 5(d)). This approach highlights community variation within each individual as a result of infection, allowing individual disease trajectories to be compared. Although unique trajectories are apparent (Figure 6), individuals showed similar longitudinal trends in phylogenetic distance of microbial lineages, which correspond negatively to the patterns of alpha diversity observed across individuals (Figure 5). Specifically, alpha diversity decreased and UniFrac distance increased during infection, suggesting that GMB membership is more depauperate and less tightly regulated than it is in the GMB of healthy sifakas.

**Table 5.** Alpha diversity across infection days in the sifakas for which initial day of infection was known.

		Shannon		Simpson		PD	
Explanatory variable	Trend	*z*	*p*	*z*	*p*	*z*	*p*
Day since infection first detected	Increasing	**2.35**	**0.019**	**2.02**	**0.043**	1.90	0.057
Age	No trend	1.31	0.190	0.78	0.436	0.95	0.343
Antimicrobials	Decreased	**−7.04**	**< 0.001**	**−6.25**	**< 0.001**	**−7.65**	**< 0.001**
Infection day * Age	No trend	−1.23	0.220	−1.32	0.187	−0.84	0.4

PD = phylogenetic diversity.

Significant results are shown in bold.

### Model 3: individual variation in gut microbiomes during infection

We used PCoA to compare samples from six infected subjects for which we had significant depth of sampling across the duration of infection and recovery (Figure 2). Based on weighted UniFrac distance (Figure 6(b)), we observe that overall trends were similar per stage across individuals, yet each sifaka’s illness progressed along a unique trajectory. Not only did each individual’s GMB occupy a unique area in vector space, but the three youngest subjects (Figure 6(b) top row) showed more variation than did the sub-adults (Figure 6(b), bottom row). As individuals recovered, their GMB regained stability. The individual distance between recovery and pre-infection baseline fell within the normal range of variation for healthy (unaffected and pre-infection) sifakas, although the recovered community was shifted from its original composition (Figures 5 and 6). This shift may be due to substitution of related species (e.g. Lachnospiraceae: other and *Lachnospira* are replaced by Lachnospiraceae: unknown and *Blautia*) or to a shift in OTU abundance.

## Discussion

This study of *Cryptosporidium* infection in the Coquerel’s sifaka suggests that infection decreases microbial diversity by acting on specific taxa, after which the GMB gradually recovers its original stable state. Thus, we infer that there are general biological forces that govern the GMB climax community, both in the case of the assembly of the original adult GMB, and in the post-disease state ‘recovery’ GMB. We found clear signals in the structure of the sifaka GMB, determined via amplicon sequencing, across infection and recovery periods. Before infection, sifakas hosted a GMB similar to that of unaffected adults. Once infected, however, GMB diversity dropped off, community distance from pre-infection baseline dramatically increased, and sifakas hosted a number of bacteria known to colonize humans with enteric disease, including *Desulfovibrio* [46], *Enterococcus*, and Enterobacteriaceae [47]. After *Cryptosporidia* oocysts cleared the lumen, the sifakas began to regain the diversity and pre-infection composition of their GMB, although these patterns were age associated, such that younger individuals recovered the most slowly. Despite our limited sample size, we observed that the initial drop in diversity and subsequent recovery from infection-induced perturbation in sifakas mirrored the initial colonization dynamics that are evident from birth to weaning [28]

Among the most notable findings in our study were those revealed by examining GMB community diversity and UniFrac distance. Diversity is generally beneficial to communities, as it probably represents more complete niche specialization and utilization, as well as functional redundancy, which can help to buffer against perturbation [48,49]. Nevertheless, because none of our diversity measures varied between unaffected and pre-infection individuals, it would seem that diversity alone may not provide resistance against *Cryptosporidium* colonization. Instead, we found that diversity measures dropped with initial infection and recovered slowly as infections cleared. These patterns during infection and recovery in lemurs are broadly consistent with what has been observed in model systems [16], and may be associated, in part, with changes in stool consistency. In a study on healthy women, researchers found that microbial richness and abundance of specific OTUs were decreased in stool samples with higher (more liquid) scores on the Bristol Stool Scale [50]. Notably, although the identity and abundance of individual taxa may have shifted, the measure of bacterial phylogenetic diversity was the least affected by infection, indicating that secondary succession may involve closely related taxa. This interpretation is further supported by measures of weighted UniFrac distance that increased during infection, relative to pre-infection baseline, but returned to values typical of healthy individuals during recovery. As in other studies [51,52], we also detected individual variation in GMB composition within sifakas. Individual trajectories during recovery could have implications for future management and treatment of enteric disease, notably if treatment options could be optimized at the individual level, rather than the species level.

In addition to identifying patterns across infection in both within- and between-sample diversity, we identified a temporal component to recolonization. *Bifidobacterium*, *Akkermansia*, *Succinovibrio*, Rickenellacaea species, and Lachnospiraceae species, which previously have been identified as commensal and/or mutualists in humans [53] and lemurs [28], were significantly enriched in healthy sifakas compared to infected or recovering individuals. Of these bacterial taxa, *Akkermansia* are also more prevalent in healthy women who have firm stools [50], suggesting that they are adapted to slow gut transit times. In addition, *Akkermansia* and *Bifidobacterium* are known mucin degraders that are depleted in humans with Crohn’s disease or ulcerative colitis [54]. Conversely, we identified six OTU biomarkers for *Cryptosporidium* infection in this study (Table 4), which may be adapted to rapid gut transit time, inflammation, and other hallmarks of disturbance. Four of these taxa have been previously described in studies of species with short gut transit times (McKenney et al. in review) or in humans with GMB dysbiosis associated with inflammatory bowel disease (IBD). For example, *Desulfovibrio* prevalence is significantly enriched in patients with IBD relative to healthy individuals or to patients with non-inflammatory bowel diseases [46]. That *Desulfovibrio* were likewise biomarkers for cryptosporidiosis in sifakas further supports the idea that sulfur-reducing bacteria may be specifically adapted to dysbiotic conditions, such as inflammation and rapid gut transit time. Like *Desulfovibrio*, *Veillonella* is significantly increased in patients with Crohn’s disease [55], and *Limnobacter* species increase significantly in a zebrafish model of IBD-like colitis [56]. Lastly, the genus *Bacillus* includes both environmental and pathogenic species [57], suggesting that the genus is an opportunistic colonizer of the sifaka gut after a microbial ‘washout’. Our results agree with previous evidence that pioneer and opportunistic microbes are well adapted to both early succession and stress [32].

Although it is unclear whether these four biomarkers directly contributed to the sifakas’ symptoms in the current study, the OTUs may be adapted to invade disrupted communities and/or guts with rapid transit times. Importantly, *Desulfovibrio* and Veillonellaceae have been implicated in the short-chain fatty acid environment of Crohn’s patients, wherein they shift their metabolism from butyrate to propionate production and increase mucin degradation [58]. The implications of such shifts are two-fold: first, a shift from butyrate to propionate production decreases the nutrients immediately available to intestinal cells; and secondly, increased mucin degradation can form toxic compounds that, in turn, may cause or perpetuate local inflammation and/or enable increased pathogen colonization [59]. The combination of effects could be especially debilitating in folivorous sifakas that depend on gut microbes to extract nutrients from a fiber-dense diet. Further research is needed to confirm whether these taxa are indeed associated with a shift in fermentation. Recovering sifakas were distinguished by only four biomarkers, the taxonomic identities of which suggest that GMBs returned to normality. For example, the presence of Lachnospiraceae species indicates a return to microbial community membership more typical of healthy sifakas, while *Coprobacillus* may have been introduced via coprophagy (a behavior typical of sifakas and other herbivorous species [60–62]) or therapeutic fecal microbiome transplant [63].

Fecal transplants, administered as treatment after infection or antibiotic perturbation, are proving to be effective in replenishing depleted communities to healthy stable states [63–65]. Following severe infection and significant antimicrobial administration, three sifakas in our study received fecal transplants from healthy donors. Although our sample size is small, these three individuals (Valeria, Gisela, and Remus) were the only subjects whose GMB diversity within the study period recovered to levels equal to or greater than that of pre-infection baseline (Figure 5(a), top row). In contrast, recovery of GMB diversity in the remaining sifakas, which received little or no antimicrobial treatment and no fecal transplant, were observed to asymptote below their pre-infection levels (Figure 5(a), bottom row). Sampling was suspended after 65 days and it is therefore conceivable that these individuals eventually recovered to their pre-infection diversity levels. That fecal transplants may have facilitated a faster recovery in sifakas is an important finding for health management. Future studies could be aimed at more carefully quantifying the functional and structural GMB patterns associated with fecal-transplant treatments in sifakas and other animals, including humans.

The constraints we faced herein, including small sample size, opportunistic sampling, and limited control of treatment administration (e.g. antimicrobials and fecal transplants), are far outweighed by the benefits gained from more broadly probing the links between the GMB and health. Because lemurs are also Earth’s most endangered vertebrates [66], studies of their GMBs may aid conservation and management efforts for wild and captive populations, respectively. In other lemur studies, a focus on diet and the GMB is helping to reveal the role of dietary fiber in maintaining gut health [28], a crucial area of research for westernized human societies [4,67]. The temporal patterns in GMB diversity and membership across infection that we observed in sifakas mirrored the dynamics observed in humans during bouts of enteric infection. Only through a comparative perspective can we appreciate the intricate symbiosis between hosts and their GMBs – information that will be crucial for addressing the health challenges of humans and wildlife.

## Supplementary Material

Supplementary material
